# Expression and Predictive Value of Serum NLR, PLR Combined with SAA in Patients with Different Stages of Colorectal Cancer

**DOI:** 10.3389/fsurg.2022.906074

**Published:** 2022-05-25

**Authors:** Qinghua Yang, Chengcheng Sun, Lisha Zhao

**Affiliations:** ^1^ Department of Anorectal Surgery, Yiwu Central Hospital of Zhejiang Province, Yiwu, China; ^2^ Department of Anesthesiology, Yiwu Central Hospital of Zhejiang Province, Yiwu, China; ^3^ Department of Medical Oncology, Zhuji People’s Hospital of Zhejiang Province, Zhuji, China

**Keywords:** colorectal cancer, neutrophil to lymphocyte ratio, platelet to lymphocyte ratio, serum amyloid A, predictive value

## Abstract

Colorectal cancer (CRC) is one of the major causes of death in the world, and has become a serious threat to human life. The prognosis of CRC patients in different pathological stages is quite different, so it is necessary to evaluate the clinical stages of CRC patients before surgery. Neutrophil to lymphocyte ratio (NLR), platelet to lymphocyte ratio (PLR), serum amyloid A (SAA) and other indicators have been widely proved to play the role of early diagnosis and prognosis monitoring in chronic inflammatory diseases and cancers. In this study, we collected clinical data of 103 patients with CRC confirmed by pathology in Yiwu Central Hospital from January 2019 to December 2021. In addition, it aims to explore the expression and predictive value of NLR, PLR combined with SAA in patients with different stages of CRC, so as to provide reference for patients to choose a reasonable treatment plan. The results show that serum NLR, PLR combined with SAA can predict CRC staging effectively, which has certain auxiliary value for clinical decision-making.

## Introduction

In 2020, colorectal cancer (CRC) is the third most common malignant tumor in the world (1.93 million), with 940,000 deaths. In 2020, CRC is the second most common malignant tumor in China (560,000), with 290,000 deaths. CRC is a serious threat to human health worldwide, and it has become a heavy global public health burden (1, 2). In recent years, medical technology has maintained rapid development, people’s understanding of CRC has been greatly improved. At present, surgery, radiotherapy, chemotherapy, molecular therapy and targeted drug therapy have made great achievements in the field of CRC. Surgical tumor resection is the fundamental method for the treatment of CRC, which greatly improves the survival rate of patients (3). However, in China, with the improvement of social level and the change of people’s living habits, the probability of CRC patients diagnosed every year is still at a high level. The prognosis of CRC patients in different pathological stages is quite different, so it is necessary to evaluate the clinical stages of CRC patients before surgery. For early-stage CRC patients, clarifying the clinical stage of the patients is helpful for formulating subsequent treatment plans and monitoring the recurrence of patients. For patients with advanced CRC, the judgment of clinical stages can improve the level of treatment and improve the treatment effect (4). Although the clinical stage of CRC patients can be determined by needle biopsy, local infiltration, lymph node metastasis and distant metastasis, these methods are traumatic (5). Therefore, in order to more comprehensively assess the prognosis, monitor the development of diseases, and improve the survival and prognosis of patients, it is clinically necessary to find more effective and minimally invasive prediction indicators to predict the stages of CRC.

Studies have shown that in tumor patients, after inflammatory mediators are released by tumor cells, they will promote the proliferation and migration of tumor cells, and inflammatory indicators may indicate tumor staging (6). As the main components of anti-inflammation and immune surveillance, the role of neutrophil (NEU), platelets (PLT) and lymphocyte count (LYM) in the occurrence and development of tumors has received increasing attention. NEU can reflect the inflammatory state of the body, and LYM can reflect the stress state of the body (7). Neutrophil to lymphocyte ratio (NLR) can reflect the systemic inflammation, and the imbalance of NLR may be related to the occurrence and development of tumors (8). Platelet to lymphocyte ratio (PLR) is a relevant marker reflecting the changes of PLT and LYM counts, which can assess the severity of infectious diseases, and also reflect or evaluate the degree of thrombosis and inflammatory response in the body. When the body’s PLR level increases, it may indicate that the body’s immune homeostasis is destroyed and the risk of tumor metastasis may increase (9). Serum amyloid A (SAA) is an acute apolipoprotein reactant, mainly produced by liver cells and regulated by inflammatory cytokines. When the body is damaged by infection, trauma, inflammatory response, cancer, etc., the serum SAA level can be significantly increased (10).

NLR, PLR, SAA and other indicators have been widely proved to play the role of early diagnosis and prognosis monitoring in chronic inflammatory diseases and cancers, but so far, there is no report on the correlation analysis between NLR, PLR, SAA and CRC patients. Therefore, we aim to discuss the expression and predictive value of NLR, PLR combined with SAA in patients with different stages of CRC, so as to provide reference for patients to choose a reasonable treatment plan.

## Materials and Methods

### Research Object

From January 2019 to December 2021, the clinical data of 103 patients with CRC confirmed by pathology in Yiwu Central Hospital were collected. Inclusion criteria: Patients underwent radical resection or palliative care for CRC after admission; The patient had not received any treatment for CRC before admission; Within 2 weeks before admission, the patients did not take anti-inflammatory, anticoagulation, glucocorticoid, immunosuppressants and other drugs. Exclusion criteria: CRC patients without surgery; Patients with signs such as acute infection, blood diseases and high fever before surgery; CRC patients with metastases from other organs; Patients with recurrence of colorectal tumors undergoing reoperation; No drugs that might have influenced the study results were administered preoperatively; Patients with severe heart and lung diseases; There were autoimmune diseases; Patients with other malignant tumors; Incomplete clinical data.

### Methods

Puncture biopsy was performed on CRC patients, the puncture path was determined, the needle was inserted to the edge of the tumor, the material was collected, and sent to the pathology department for pathological examination. CRC specimens were divided into 4 stages according to TNM stage (11): I-II, III and IV. As the stage increases, the symptoms of cancer also worsen.

When the patient was admitted to hospital, 4 mL of elbow venous blood was taken in the morning, centrifuged for 15 min, and the upper serum was taken and frozen in the refrigerator, prepared for subsequent use. PLT was detected by direct current impedance method of sheath fluid, and white blood cells (NEU, LYM) were detected by fluorescence staining flow cytometry. Automatic blood cell analyzer (Sysmex XE-5000) was used to test the blood within 2 h after blood collection. NLR = NEU/LYM、PLR = PLT/LYM. SAA was detected by immune timing nephelometry, and detected by automatic biochemical analyzer (BECKMAN DxI 800) within 2 h after blood collection. Tests were carried out in strict accordance with the requirements, and the quality control of the project was carried out.

### Statistical Methods

SPSS 22.0 was used to process the data. Measurement data conforming to a normal distribution was expressed by mean ± standard deviation. ANOVA test was used for comparison among multiple groups, and SNK-q test was used for pairwise comparison. Counting data was expressed by ratio, and χ^2^ test was used for pairwise comparison. ROC curve was drawn, and AUC was used to predict the diagnostic value of serum NLR, PLR and SAA levels in staging of CRC patients. AUC value >0.7 had good predictive value. *p* < 0.05 was statistically significant.

## Results

### Basic Information of CRC Patients in Different Stages

Among 103 CRC patients, 22 cases (21.36%) were in stage I–II, 49 cases (47.57%) were in stage III and 32 cases (31.07%) were in stage IV. There was no significant difference in age, gender and body mass index among CRC patients in different stages (*p* > 0.05). See Table 1.

**Table 1:** Basic information of CRC patients in different stages.

Group	Age (years)	Gender		Body mass index (kg/m^2^)
		Male	Female	
Stage I-II (*n* = 22)	65.94 ± 4.25	14 (63.64%)	8 (36.36%)	23.91 ± 3.17
Stage III (*n* = 49)	66.16 ± 4.28	33 (67.35%)	16 (32.65%)	24.62 ± 3.04
Stage IV (*n* = 32)	68.20 ± 4.57	24 (75.00%)	8 (25.00%)	24.34 ± 3.09
*χ^2^/t value*	2.591	0.896		0.406
*p value*	0.080	0.639		0.667

### Serum NLR, PLR and SAA Levels of CRC Patients in Different Stages

**Figure 1:**
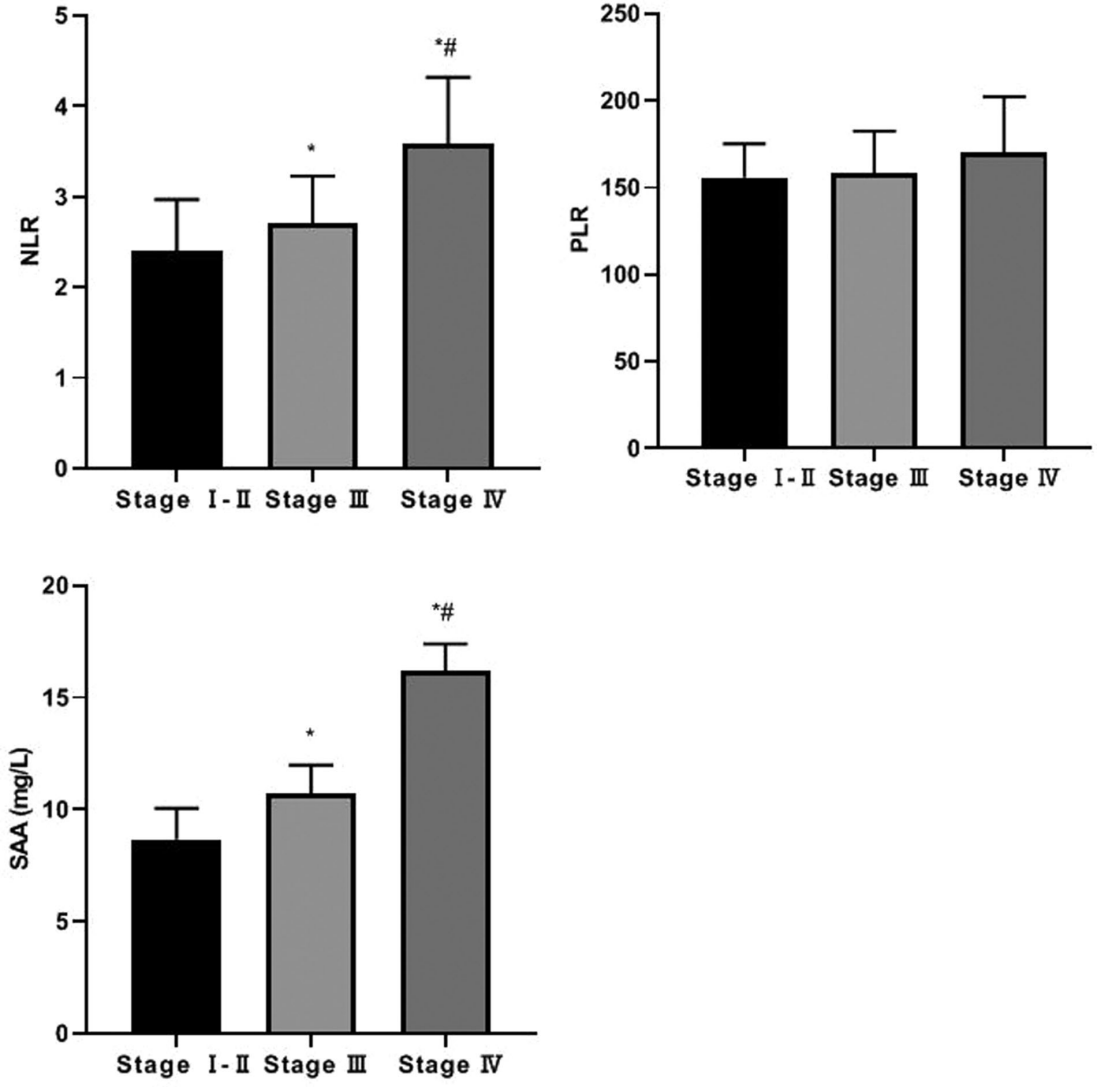
Serum NLR, PLR and SAA levels of CRC patients in different stages Compared with Stage I–II, *p < 0.05; Compared with stage III, #*p* < 0.05.

The levels of serum NLR and SAA in patients with stage IV were higher than those in patients with stage I–II and stage III, and those in patients with stage III were higher than those in patients with stage I–II (F = 30.937, F = 264.814, *p* < 0.05). There was no significant difference in serum PLR levels among CRC patients in different stages (F = 2.7986, *p* > 0.05). See Figure 1.

### Value of Serum NLR, PLR and SAA Levels in Predicting Patients with Stage I-II CRC

**Figure 2:**
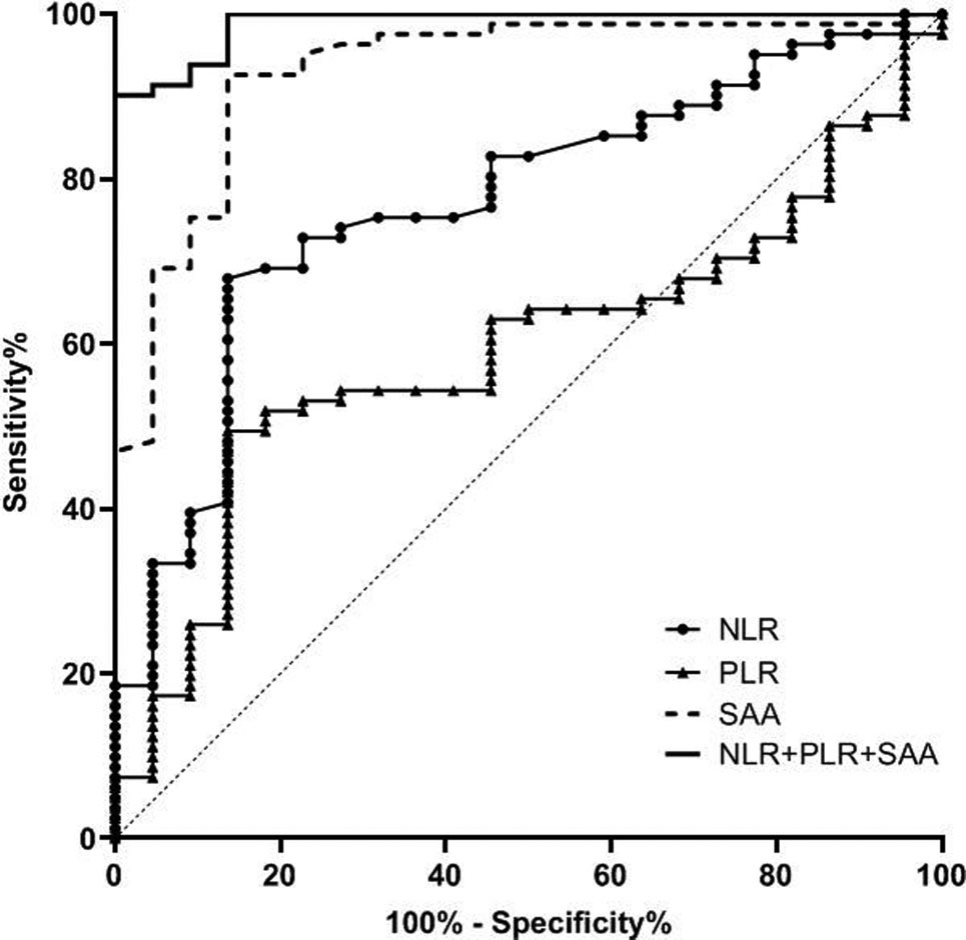
Value of serum NLR, PLR and SAA levels in predicting patients with stage I-II CRC.

The AUC of serum NLR, PLR and SAA levels in predicting CRC patients with stage I-II was 0.770, 0.593 and 0.931 respectively, and the AUC of combination of the three in predicting CRC patients with stage I-II was 0.989. See Table 2 and Figure 2.

**Table 2:** Value of serum NLR, PLR and SAA levels in predicting patients with stage I–II CRC.

Variable	AUC	Standard error	*p* value	Asymptotic 95% CI	
				Lower limit	Upper limit
NLR	0.770	0.054	0.000	0.665	0.875
PLR	0.593	0.060	0.184	0.474	0.711
SAA	0.931	0.031	0.000	0.871	0.991
NLR + PLR + SAA	0.989	0.008	0.000	0.974	1.000

### Value of Serum NLR, PLR and SAA Levels in Predicting Patients with Stage III CRC

**Figure 3:**
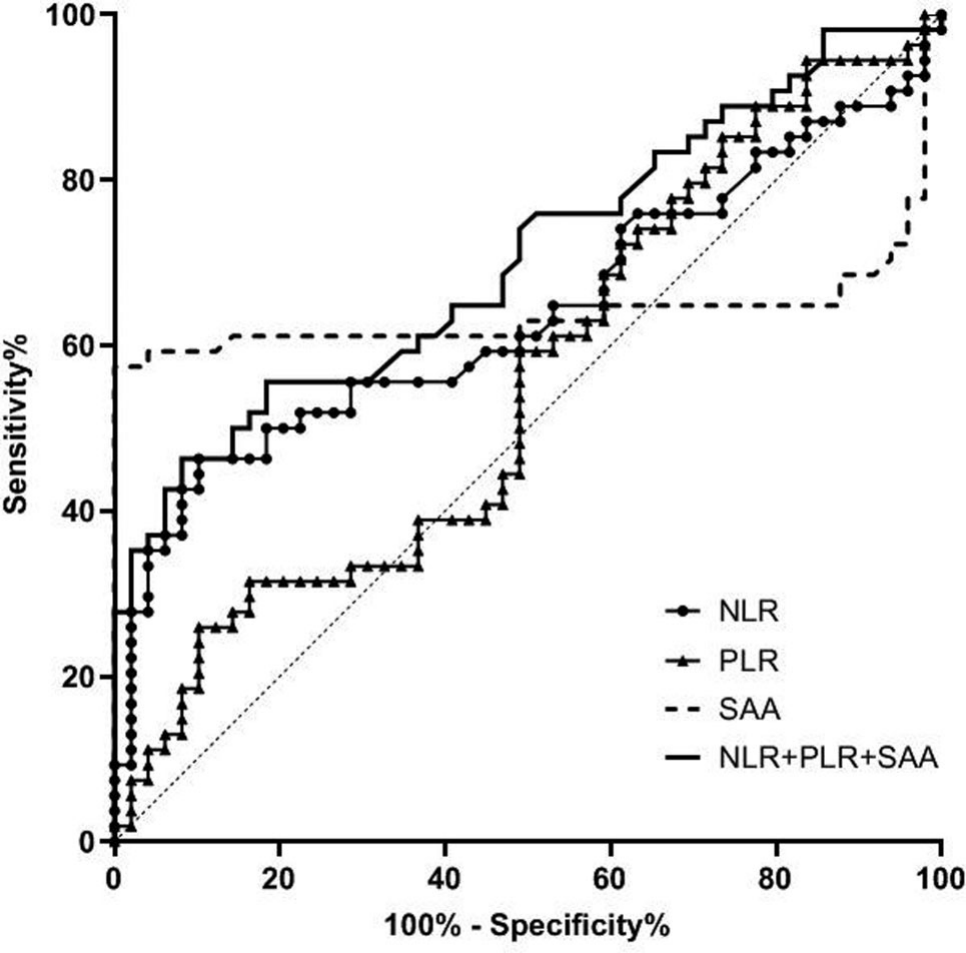
Value of serum NLR, PLR and SAA levels in predicting patients with stage III CRC.

The AUC of serum NLR, PLR and SAA levels in predicting CRC patients with stage III was 0.640, 0.556 and 0.638 respectively, and the AUC of combination of the three in predicting CRC patients with stage III was 0.706. See Table 3 and Figure 3.

**Table 3:** Value of serum NLR, PLR and SAA levels in predicting patients with stage III CRC.

Variable	AUC	Standard error	*p* value	Asymptotic 95% CI	
				Lower limit	Upper limit
NLR	0.640	0.055	0.015	0.531	0.748
PLR	0.556	0.057	0.325	0.444	0.668
SAA	0.638	0.063	0.016	0.516	0.761
NLR + PLR + SAA	0.706	0.051	0.000	0.607	0.806

### Value of Serum NLR, PLR and SAA Levels in Predicting Patients with Stage IV CRC

**Figure 4:**
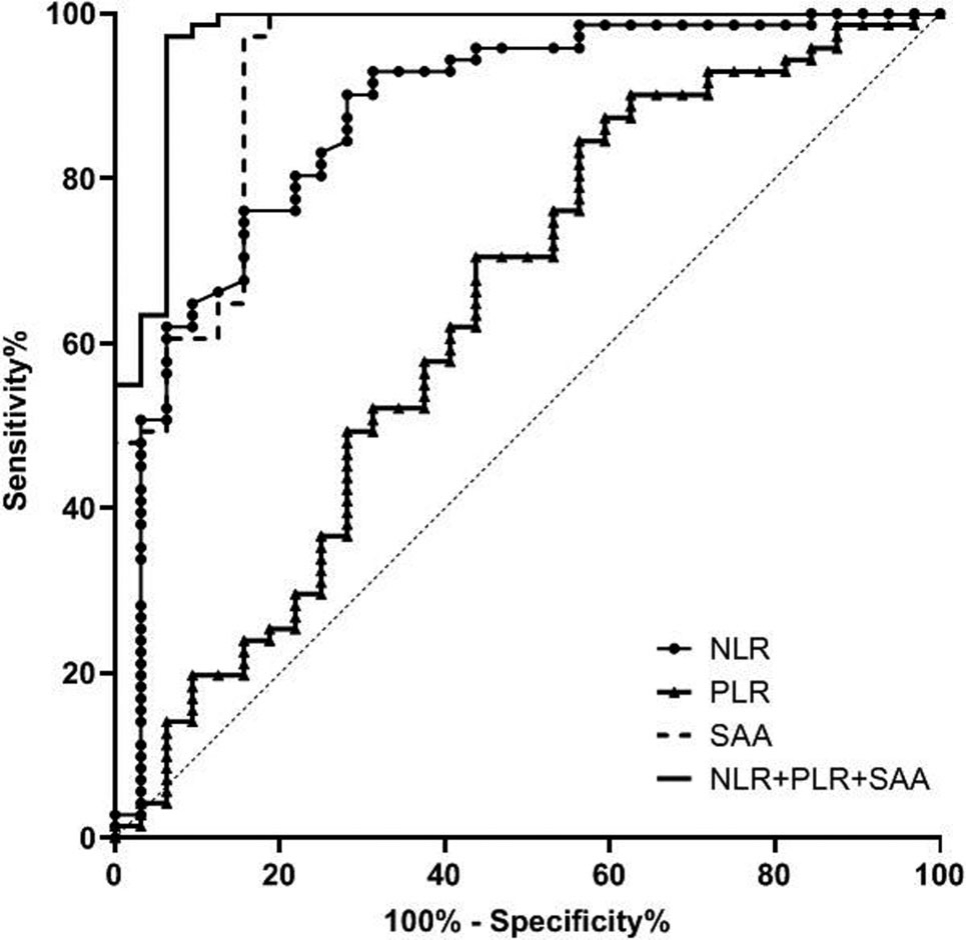
Value of serum NLR, PLR and SAA levels in predicting patients with stage IV CRC.

The AUC of serum NLR, PLR and SAA levels in predicting CRC patients with stage IV was 0.874, 0.638 and 0.931 respectively, and the AUC of combination of the three in predicting CRC patients with stage IV was 0.973. See Table 4 and Figure 4.

**Table 4:** Value of serum NLR, PLR and SAA levels in predicting patients with stage IV CRC.

Variable	AUC	Standard error	*p* value	Asymptotic 95% CI	
				Lower limit	Upper limit
NLR	0.874	0.039	0.000	0.796	0.952
PLR	0.638	0.062	0.025	0.515	0.761
SAA	0.931	0.030	0.000	0.873	0.989
NLR + PLR + SAA	0.973	0.018	0.000	0.937	1.000

## Discussion

CRC is one of the major causes of death in the world, and has become a serious threat to human life. In recent years, despite the rapid development of surgery, chemotherapy and molecular therapy, the prognosis of CRC patients is not good due to the fact that tumors tend to be deeper local infiltration, lymph node metastasis, and some patients are accompanied by cardiovascular diseases and immune system diseases (12). Since the staging system is related to the prognosis of CRC patients, it is necessary to find serum biological indicators that can effectively assess tumor staging, and research in this area has received more and more attention.

NEU is the white blood cell with the largest content in peripheral blood, accounting for 40%–60% of leukocytes. NEU is mainly involved in innate immunity, that is, nonspecific inflammatory reaction (13). NEU plays an key role in host protection against infection and inflammation. It can cause tissue damage, provide signals to epithelial cells, macrophages and other immune cells, and then increase LYM, thus regulating the subsequent inflammatory response (14). NEU promotes the formation of blood vessels and tumor growth by secreting vascular growth factors; It is also possible to accelerate tumor progression by promoting the release of proteins such as epidermal growth factor and platelet-derived growth factor in extracellular matrix (15, 16). Granot’s team found that some NEU can kill tumor cells through direct cytotoxicity or antibody-dependent cell-mediated cytotoxicity (17). LYM accounts for 20%–40% of peripheral white blood cells, which is an important cell of human immune response and participates in adaptive immunity. LYM is an important part of anti-tumor immunity, which plays an immune surveillance role. When the body suffers from severe immune dysfunction and immunosuppression, it will cause a series of pathophysiological changes (18). LYM can specifically recognize and directly kill tumors or release a series of cytokines to activate anti-tumor immunity, which plays a key role in the production of cytokines that inhibit tumor proliferation and metastasis (19). NEU and LYM are objective indicators reflecting the inflammatory response and immune status of the body. However, a single indicator is greatly affected by physiology and stress, and the detection results have a large fluctuation range and low stability. NLR can stably reflect the relationship between multiple factors and the interaction of inflammatory response, immune system in the body (20). In this study, CRC staging is positively correlated with NLR. The possible reasons are as follows: with the increase of stage, the body’s inflammatory response increases, and the level of NEU in CRC patients increases. At the same time, there may also be a decrease in the body’s immune function and LYM level, so NLR increases. With the increase of CRC staging and the deepening of tumor invasion, lymph node metastasis and distant metastasis may occur, thus stimulating the growth-promoting effect of NEU on tumor and reducing anti-tumor cellular immune response mediated by LYM. NLR has the characteristics of convenient sampling, low cost, simplicity and rapidity, and can be used as an indicator to observe systemic inflammatory response.

PLT plays an key role in the regulation of hemostasis, wound healing, inflammatory response, immune response and tumor development (21). After tumor cells are detected, PLT will be rapidly activated, and start to drive the inflammatory response, directly regulate the activity of NEU, LYM and endothelial cells, and promote the aggregation of various adhesion molecules and chemokines to the tissue injury site. In addition, PLT is involved in the growth and metastasis of tumor cells by releasing platelet-derived growth factors and many angiogenic proteins. At the same time, tumor cells can induce PLT aggregation and manipulate PLT activity to promote tumor progression (22). PLR can be used to assess the dynamic balance between anti-tumor immunity in vivo. When PLR is high, there is an immune imbalance between anti-tumor and tumor-promoting (23). Catal’s team research shows that PLT supports the formation of blood vessels in tumor tissues, the growth and diffusion of cancer cells and subsequent secondary lesions; PLR is related to the size and degree of tumor invasion (24). However, some reports suggest that PLR is not an independent predictor of CRC staging (25). Our study also found that there was no significant difference in serum PLR levels among patients with different stages of CRC, and the AUC of PLR in predicting CRC patients with different stages was smaller. Therefore, the value of PLR in predicting CRC in different stages is still controversial, which requires scholars to conduct further experiments to explore the reasons.

SAA is a substance synthesized and induced by tumor necrosis factor, interleukin −1 and interleukin −6. When the body is stimulated by inflammation or infection, the serum levels of SAA can increase abnormally (26). Davis’ team reported that the progress and metastasis of CRC are closely associated with inflammatory response (27). It is speculated that the detection of serum SAA level may play a certain role in predicting the clinical stage of CRC patients. In this study, with the increase of CRC stage, the level of serum SAA increased. The results suggest that SAA has certain evaluation value for the staging of CRC patients. As an inflammatory factor, SAA can participate in the process of inducing gene mutation, change the tumor microenvironment and promote the occurrence and development of tumors. In addition, the synthesis of SAA is regulated by a variety of inflammatory factors, and the up-regulation of these factors can further promote tumor proliferation and accelerate tumor progression.

In addition, the ROC curve results of this study found that the AUC of serum NLR, PLR and SAA levels in predicting CRC staging was higher, and the AUC of combination of the three in predicting CRC patients with stage I-II, stage III and stage IV was 0.989, 0.706 and 0.973 respectively. Compared with a single detection index, the AUC of NLR, PLR combined with SAA in predicting CRC staging was significantly higher. This further indicates that the combined detection of NLR, PLR and SAA can effectively evaluate the clinical stage of CRC patients and make up for the defect of low sensitivity of single marker to CRC. Therefore, the detection of NLR, PLR combined with SAA can provide some auxiliary value for the surgical preparation and prognosis of CRC patients.

## Conclusion

To sum up, serum NLR, PLR combined with SAA can predict CRC staging effectively, which has certain auxiliary value for clinical decision-making. This study has the following shortcomings: it is a retrospective study, a small sample size, a single-center trial, and there is potential selection bias. In addition, this study only considered the serum NLR, PLR, and SAA levels of CRC patients on admission, and did not monitor the changes of these indicators in patients who underwent radical surgery. We need to further improve the research plan in the future.

## Data Availability

The original contributions presented in the study are included in the article/Supplementary Material, further inquiries can be directed to the corresponding author/s.
